# Improving Foot Care in Psychiatric In-Patient Rehabilitation: Introducing Structured Foot Checks

**DOI:** 10.1192/bjo.2026.11375

**Published:** 2026-06-30

**Authors:** Chinenye Ezemagu-Onedi

**Affiliations:** Devon Partnership NHS Trust, Exeter, United Kingdom

## Abstract

**Aims:**

This quality improvement project aimed to assess current foot care practices on a psychiatric rehabilitation ward and to inform the implementation of a structured approach to routine foot checks delivered by ward staff.

**Methods:**

A cross-sectional study with semi-structured questionnaire administered by physicianto in-patients on a psychiatric rehabilitation ward to assess foot care practices in December 2025. Data were collected on demographics (age, sex), length of admission, history of foot inspection during admission, presence of foot problems, treatment received, patient attitudes towards routine foot checks, and risk factors for foot-related complications. Eight patients completed the questionnaire; two patients were unable to participate due to their current mental state. There were no exclusion criteria.

**Results:**

Only 25% of patients reported having had their feet inspected since admission. Of those who received a foot check, one patient was referred to podiatry for further assessment.

50% of these patients fall within the age of 45–64years.

75% of these patients have been on the current ward for more than a month and none expressed a preference for regular foot checks by staff. Three patients were identified ashaving risk factors for developing foot-related problems, including medical comorbidities and lifestyle factors, without evidence of consistent monitoring.

88% of these patients haven’t experienced any foot conditions since their stay.

**Conclusion:**

Routine foot care assessment was infrequent despite identifiable patient risk factors. Introducing a structured foot-check protocol with clearly defined staff responsibility may improve early identification of foot problems and support preventative physical health care in psychiatric rehabilitation settings. No external financial sponsorship was received for this project.

